# Synthesizing efficacious genistein in conjugation with superparamagnetic Fe_3_O_4_ decorated with bio-compatible carboxymethylated chitosan against acute leukemia lymphoma

**DOI:** 10.1186/s40824-020-00187-2

**Published:** 2020-03-20

**Authors:** Rachel Ghasemi Goorbandi, Mohammad Reza Mohammadi, Kianoosh Malekzadeh

**Affiliations:** 1 Sharif University of Technology, Kish International Campus, Kish Island, Iran; 2 Molecular Medicine Research Center Health Institute, Hormozgan University of Medical Sciences, Bandar Abbas, Iran; 3 Department of Materials Science and Engineering, Sharif University of Technology, Tehran, Iran; 4 Department of Medical Genetics; Faculty of Medicine, Hormozgan University of Medical Sciences, Bandar Abbas, Iran

**Keywords:** Acute leukemia lymphoma (ALL), Chitosan, Genistein, Superparamagnetic Fe_3_O_4_, Nano particle delivery system

## Abstract

**Background:**

Genistein (C_15_H_10_O_5_) is a soy isoflavone with anti-cancer properties such as inhibition of cell growth, proliferation and tumor invasion, but effective dosage against hematopoietic malignant cells was not in non-toxic range. This property cause to impede its usage as chemotherapeutic agent. Therefore, this hypothesis raised that synthesizing biocompatible nanoparticle could assist to prevail this struggle.

**Methods:**

Genistein covalently attached on Fe_3_O_4_ nanoparticles decorated with carboxymethylated chitosan to fabricate Fe_3_O_4_-CMC-genistein in alkaline circumstance. This obtained nanoparticles were evaluated by TEM, DLS, FTIR, XRD and VSM and its anti-cancer effect by growth rate and MTT assays as well as flow cytometer on ALL cancer cell lines.

**Results:**

Different evaluations indicated that the drug delivery vehicle had a mean diameter size around 12ƞm with well bounded components. This system presented high degree of magnetization and superparamagnetic properties as well as good water solubility. In comparison with pure genistein, significant growth inhibition on hematopoietic cancer cells in lower dose of genistein nano-conjugated onto Fe_3_O_4_-CMC. It increased long lasting effect of genistein in cancer cells also.

**Conclusion:**

This delivery system for genistein could be remarkably promised and futuristic as biocompatible chemotherapeutic agent against hematopoietic malignant cells.

## Introduction

Nanoparticles are used in management of different cancers through active development for in vivo imaging for cancers, detection of tumor cells biomarkers, and usage in targeted drug delivery [1].

Magnetic nanoparticles (MNPs) are widely used for therapeutic and diagnostic objectives, because different polymer can be loaded on these materials. Another point is their ease of preparation and strong magnetic properties, which make it possible to apply them for targeted drug delivery [2]. These magnetic particles are also used for cell label and separation, immunoassay, imaging, and targeted drug delivery [3]. Targeting of specific tissues is possible by using magnetic nanoparticles due to their high magnetization value, water dispersion. These improve their non-toxic properties and biocompatibility [4].

Magnetic gene and drug delivery is one of the most important applications of iron oxide nanoparticles [5]. Superparamagnetic Iron Oxide Nanoparticles (SPIONs) are iron nanoparticles, which are widely used because of their biocompatibility and their ease of synthesis [6]. Magnetite (Fe_3_O_4_) and maghemite (γFe_2_O_3_) are the most commonly studied iron oxide particles.

For Surface stabilization of the magnetic nano-particles (MNPs), the magnetic core must be covered with organic materials and targeting ligands. As magnetic nanoparticles tend to aggregate because of their high surface energy, by using this technique, the surface of the particles will be prevented from oxidation and provide a site for linkage of drug molecules or gene vectors [5]. The core size of SPIONs ranges from 10ηm to 100ηm which makes is possible to use them in targeted drug delivery and other applications in biomedical sciences. These iron NPs can be coated by different organic and inorganic materials, which can be guided to the target tissues by their magnetic properties. Absence of magnetic properties after removal of external magnetic field avoids their agglomeration. By this mechanism, they remain in circulation and vessel embolism, therefore thrombosis is prevented [7].

Chitosan is derived from chitin (a natural polysaccharide), which is a carbohydrate found in crustaceans exoskeleton. Three different chitosan derivatives are obtained by its carboxymethylation including O-carboxymethyl chitosan, N-carboxymethyl chitosan, and N, O-carboxymethyl chitosan [8]. O-carboxymethyl chitosan (OCMCS) is widely applied in biomedical applications because of its blood compatibility properties [9]. One of the important activities of chitosan is antioxidant activity because of its activity against superoxide anion. The antioxidant activity of chitosan is used in pharmacy for production of extended-release tablet with high antioxidant activity [10]. Although chitosan is reported to induce apoptosis, but carboxymethylated chitosan (CMC) can inhibit apoptosis. It seems that it is related to the protection of mitochondrial function and decreasing nitric oxide and reactive oxygen species level [11]. Chitosan has also been known as low toxicity chemotherapeutic material, but its use is limited because of specific properties such as poor solubility in pH upper than 6.5. Chitosan is soluble only in pH < 6.5 due to strong hydrogen bands which cause stable crystalline structure. To achieve the best results and to overcome these limitations, carboxymethylated chitosan, which is soluble in alkaline and acidic aqueous solutions [8], can be attached to Fe_3_O_4_ nanoparticles to be used in drug delivery systems [12]. Different molecules and antibodies can be loaded into Fe_3_O_4_-CMC nanoparticles. In the other hand, its amphiphilic properties are helpful for its usage in biomedical researches [13].

Genistein (C_15_H_10_O_5_) is a soy isoflavone, which is interested for its anti-cancer activities and can be considered in chemotherapy [14–16] Different mechanisms of action are reported for genistein. One possible mechanism is inhibition of topoisomerase II, which decreases cell proliferation [17]. It was observed that genistein can inhibit of tumor cells in gastric, breast, lung, prostate, pancreatic, liver, ovarian, colon, and bladder cancer [15, 16]. Genistein not only prevents tumor cell growth, invasion, and metastasis but also increases tumor cells sensitivity to chemotherapy. Several studies have tried to reveal possible mechanisms of action of genistein in gastric cancer [17–19].

In gastric cancer, genistein decreases Gli1 gene expression and attenuates cancer stem-like properties. By this mechanism, genistein prevents invasion of tumor cells and inhibits tumor growth and metastasis [17]. Genistein also reduces gastric cancer chemo-resistance [18]. Another mechanism is increase in tumor suppression PTEN expression [20]. In addition to above studies, some cohort studies have reported the decrease risk of gastric cancer with isoflavones consumption [21].

As the anti-cancer feature of genistein has already been noticed as candidate herbal chemotherapeutic agent in different cancer but its effect in that growth inhibition was linearly related to genistein concentration. Carlo-Stella et al., (1996) revealed that (i) genistein strongly inhibits the growth of normal and leukaemic haemopoietic progenitors; (ii) growth inhibition is dose- and time-dependent; (iii) leukaemic progenitors are more sensitive than normal progenitors to genistein-induced growth inhibition [22]. This project was conducted to investigate whether anti-carcinogenic effects of genistein can be improved by using magnetic nanoparticles composed of a magnetic core and a biocompatible polymeric shell and genistein molecule in lower dose on hematopoietic cancer cell line, which leads to development of a promising carrier and targeting anticancer therapy.

## Material and methods

The required materials were Iron (II) chloride tetrahydrate (99%), iron (III) chloride hexahydrate (98%), ammonia solution 25% GR for analysis, (Merck, Germany)(3-Dimethylaminopropyl)-3-ethylcarbodiimide (97%), Chitosan with molecular weight of 600,000–800,000 (Acros Organices) Monochloroacetic acid, synthetic Genistein (99%), NHS (N-Hydroxysuccinimide), DMSO (Dimethyl sulphoxide), MTT (Thiazolyl Blue Tetrazolium Bromide), Annexin V-FITC Apoptosis Detection Kit (Sigma Aldrich), FBS (Fetal bovine serum), Penstrep (Penicillin /Streptomycin), RPMI-1640, PI (Propidium Iodide Solution) (GIBCO). MOLT-4 cell lines were purchased from Pasteur Institute of Iran.

### Synthesis of superparamagnetic Iron oxide nanoparticles

Magnetite nanoparticles were synthesized by coprecipitating iron (II) chloride and iron (III) chloride in alkaline solution. First 1.2 g of ferric chloride dissolved in 60 ml distilled water and ferrous solution prepared by dissolving 0.6 g of ferrous chloride in 30 ml of distilled water. After mixing of the solutions in a three neck flasks with magnetic stirring and purging the nitrogen gas for 10 min, 4 ml of 25% (w/w) NH_3_·H_2_O was added drop wise into the mixture solution and the pH of the solution was carefully monitored in order to reach 11.0. Change the color of the solution to dark black, showing formation of Fe_3_O_4_ and SPION precipitation. The black precipitate was separated from the solution with a neodymium magnet and rinsed several times with distilled water and ethanol and the pH value descended to 7.0. Resulted SPION dried in a vacuum oven in 70 °C for 4 h [23].

$$ {\mathrm{Fe}}^{2+}+\kern0.5em 2{\mathrm{Fe}}^{3+}+8\mathrm{OH}-\to {\mathrm{Fe}}_3{\mathrm{O}}_4\downarrow +4{\mathrm{H}}_2\mathrm{O} $$

### Synthesis of carboxymethyl chitosan

For synthesis of O-carboxymethyl chitosan, 3 g chitosan was immersed in 25 ml of 50% wt NaOH solution to swell and alkalize for 24 h at room temperature. The alkalized chitosan was filtered using a G2 sintered funnel. The solution of 5 g of monochloroacetic acid in 25 ml of isopropanol added drop-wise on it for 30 min. After 4 h reaction at 60 °C temperature, the solvent was removed by filtering the mixture. The obtained product was dissolved in 100 ml of distilled water and 2.5 M HCl was used to adjust the pH to 7.0. Then this solution was filtered and carboxymethylated chitosan was precipitate by adding 400 ml of anhydrous ethanol. The final product was filtered, rinsed several times with ethanol and vacuum-dried at room temperature.

### Preparation of Fe3O4 bound carboxymethylated chitosan

For preparation of carboxymethyl chitosan coated iron oxide nanoparticles, 28.25 μl of EDC (density 0.885 g/cm^3^) was added to 4 ml of phosphate buffer (0.2 mol/L Na_2_HPO_4_–NaH_2_PO_4_, pH 6.0), then 75 mg of Fe_3_O_4_ nanoparticles were added to the mixture and ultrasonicated for 10 min. After that 25 mg of carboxymethyl chitosan in 1 ml phosphate buffer was added to the obtained mixture and it was ultrasonicated for another 1 h. For distinction of the product from the mixture, neodymium magnet was used. Finally the product was washed with water and ethanol three times and vacuumed dried at room temperature.

### Immobilizing genistein onto the CMC modified Fe_3_O_4_ nanoparticles

The next step was immobilization of genistein onto chitosan coated iron oxide nanoparticles. To reach this aim, genistein (2.5 mg) EDC (5.65 μl), NHS (6 mg) were dissolved in 5 ml of phosphate buffer (pH = 6.0, 2 mmol/L). Then 10 mg of Fe_3_O_4_-CMC nanoparticles was added. The suspension was then ultrasonicated for 10 min in 4 °C and shacked at room temperature for 24 h. Fe_3_O_4_-CMC-genistein was recovered by magnet from the reaction mixture. The precipitate was washed with 2 mM phosphate buffer (pH 6.0) and vacuumed dried at room temperature.

### Characterization

Powder X-ray diffraction were recorded using a diffractometer (XRD-STOE-Stidy-mp) in 25 °C using CuKα radiation (λ = 1.54178 Å). Fourier transform infrared (FT-IR) spectroscopy of the samples was performed on a Bruker-Veator-22, FT-IR spectrophotometer for characterization the surface reaction of samples over the range of 400–4000 cm^− 1^.Morphology, mean particle size and size distribution was performed using Zeiss-EM10C, transmission electron microscopy (TEM), electron microscope operated at 100 KV DLS analysis. Magnetic characteristics were measured on a 7400 Lake shore vibrating sample magnetometer (VSM) at room temperature.

### Cell culture

MOLT-4, MOLT17 and Jurket cell lines purchased from Pasteur Institute of Iran, centrifuged (130 g for 5 min) and suspended in RPMI 1640 supplemented with 10% fetal bovine serum (FBS), 100 mg/mL streptomycine, and 100 U/mL penicillin then cultured in 6-well micro plates (9.6 cm^2^) with concentration of 15 × 10^4^ cells/ml and incubated in a humidified incubator by standard cell culture conditions (37 °C and 5% CO_2_).

### Proliferation and MTT studies

In order to conduction the inhibiting effect of nano-conjugated Fe_3_O_4_-CMC-genistein in comparison with genistein and un-treated cells, briefly, a density of 15 × 10^4^ /ml cells were seeded per each well of a six-well plate. Cells were treated with only genistein, Fe_3_O_4_-CMC nano-particles, and Fe_3_O_4_-CMC-genistein nano-conjugated with different doses from 20 and 40 μmol/L and incubated at 37 °C and 5% CO_2_. Doses 20 and 40 μmol/L selected in base of the IC_50_ obtained from MTT assay. After first 48 h, post treatment with 24 h’ interval were done for next remain 5 days. Hematocytometer was applied to count viable cells from three wells per dose. The appearance of healthy looking, rounded cells with intact cell membranes considered as viable cells versus dead or dying cells with ghostly, necrotic appearances and disrupted cell membranes. Tryphan-blue test was also performed.

Hematopoietic cancer cells were also treated with nano-conjugated Fe_3_O_4_-CMC-genistein as well as naked genistein in concentrations of 20, 40, 60, 80 and 100 μmol/L and then viable cells counted at 24, 48 and 72 h. Percent reduction of growth was calculated by dividing the number of treated cells with genistein or nano-conjugated Fe_3_O_4_-CMC-genistein to number of untreated cells.

MTT assay was also performed to detect the effect of Fe_3_O_4_-CMC-genistein nano-conjugate on proliferation of hematopoietic cancer cells as compare to naked genistein. Briefly, 20 μl MTT (5 mg/ml) was added up to each well of incubated cells with only genistein, Fe_3_O_4_-CMC nano-particles, and Fe_3_O_4_-CMC-genistein nano-conjugated, and then incubated for 4 h. Fe_3_O_4_-CMC nano-particles without genistein used as control the cytotoxic effect of nonao-particles. Then, the supernatants were removed and proceed by adding of 200 μl DMSO to dissolve formazen crystals. After 10 min incubation, the plate was read at 490 nm by micro plate reader versus to non-treated cells. Since, DMSO is solvent of genistein, and the final DMSO concentration n the medium was lesser than 0.1%, therefore, the cells treated with only DMSO also analyzed as control of cytotoxicity effect. Data of MTT assay was repeated 3 times for each cell line per dose.

### Flow cytometry

Apoptosis particularly early apoptosis was also evaluated by flow cytometry analysis. For this purpose, hematopoietic cancer cells were cultured and treated with only genistein, synthesized nanoparticles Fe_3_O_4_-CMC without geneistein, and synthesized nano-conjugated Fe_3_O_4_-CMC-genistein for 48 h. Then, ice-cold PBS used to resuspend in 500 μL binding buffer and then incubated for 25 min more in dark. After that, solution of annexin-V and propidium iodide (PI) was added to the cells and then 400 μl binding buffer was added to each tube. FACS Calibur (BD; USA) instrument was used for analyzing.

### Statistical analysis

**Fig. 1:**
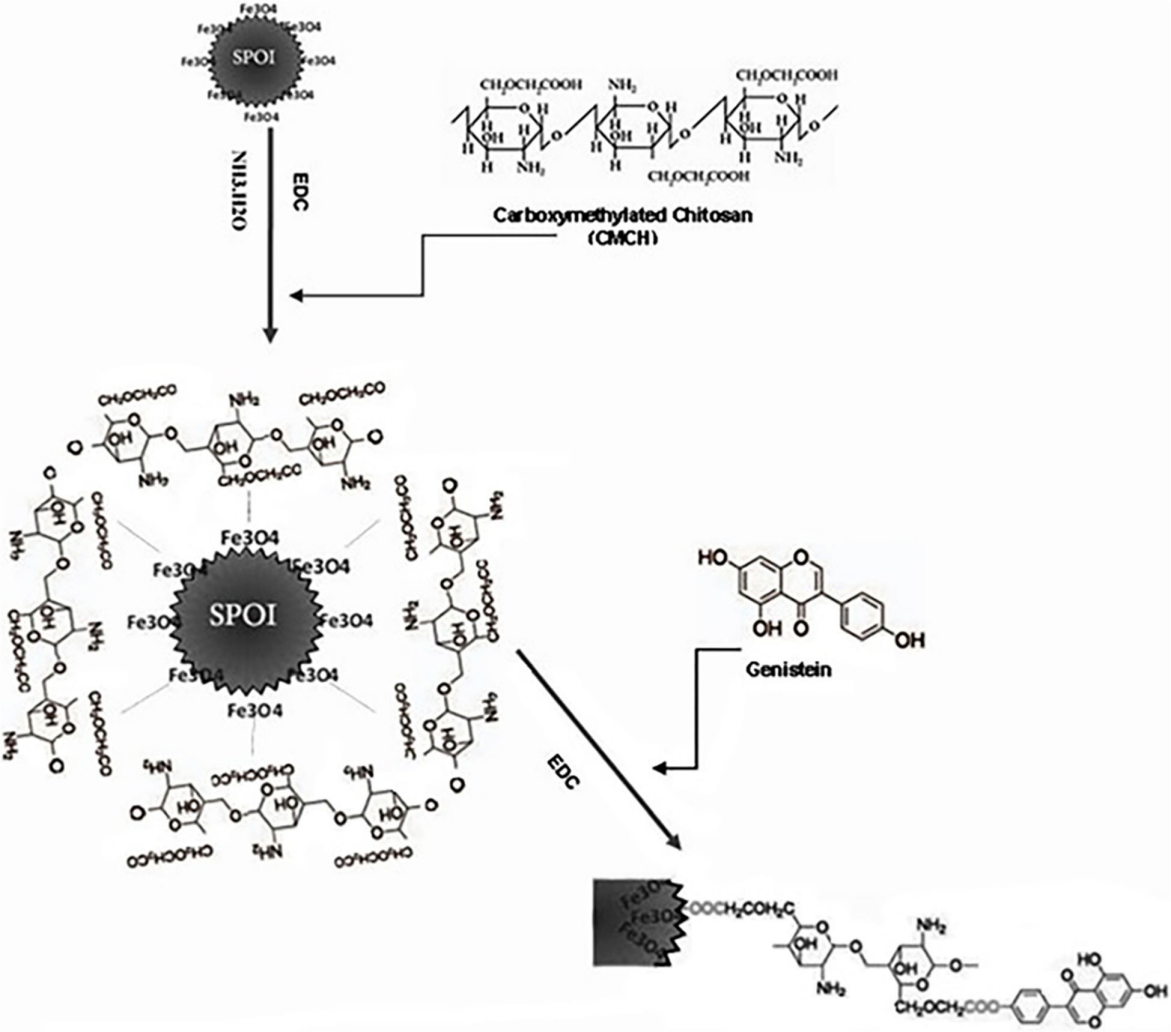
Schematic illustration for synthesizing Fe3O4-CMCH-genistein nanoparticles

The data were analyzed using Excel 2010, Graph Pad Prism 5.0 and SPSS softwares. Comparison of growth rate, apoptosis of cancer cell line between non-treated and treated, and nano-conjugated and naked genistein in different doses were performed by the non-parametric test followed by Mann-Whitney test and *p*-values < 0.05 were considered as statistically significant. Figure 1 shows a schematic illustration of synthesis methods and cell treatment.

## Results

### Particle size and morphology of Fe_3_O_4_ and Fe_3_O_4_-CMC-genistein nanoparticles

**Fig. 2:**
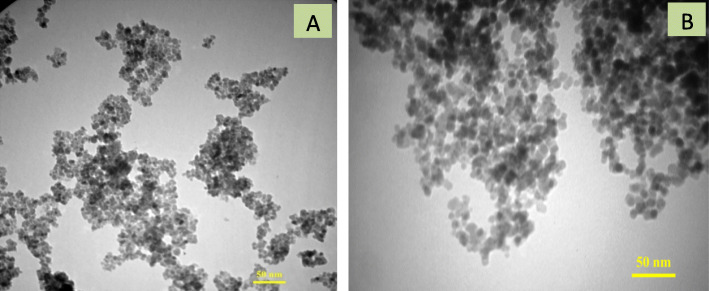
TEM micrographs of (**a**) Iron oxide nanoparticles (Fe3O4 NPs) and (**b**) Genistein conjugated Fe3O4-CMC nanoparticles

For confirmation of the size and morphology of Fe_3_O_4_nanoparticles and the Fe_3_O_4_-CMC-genistein samples, transmission electron microscopy (TEM) was used. As shown in Fig. 2, the iron oxide nanoparticles and Fe_3_O_4_-CMC-genistein particles had spherical shape and were distanced from each other and tend to aggregate.

**Fig. 3:**
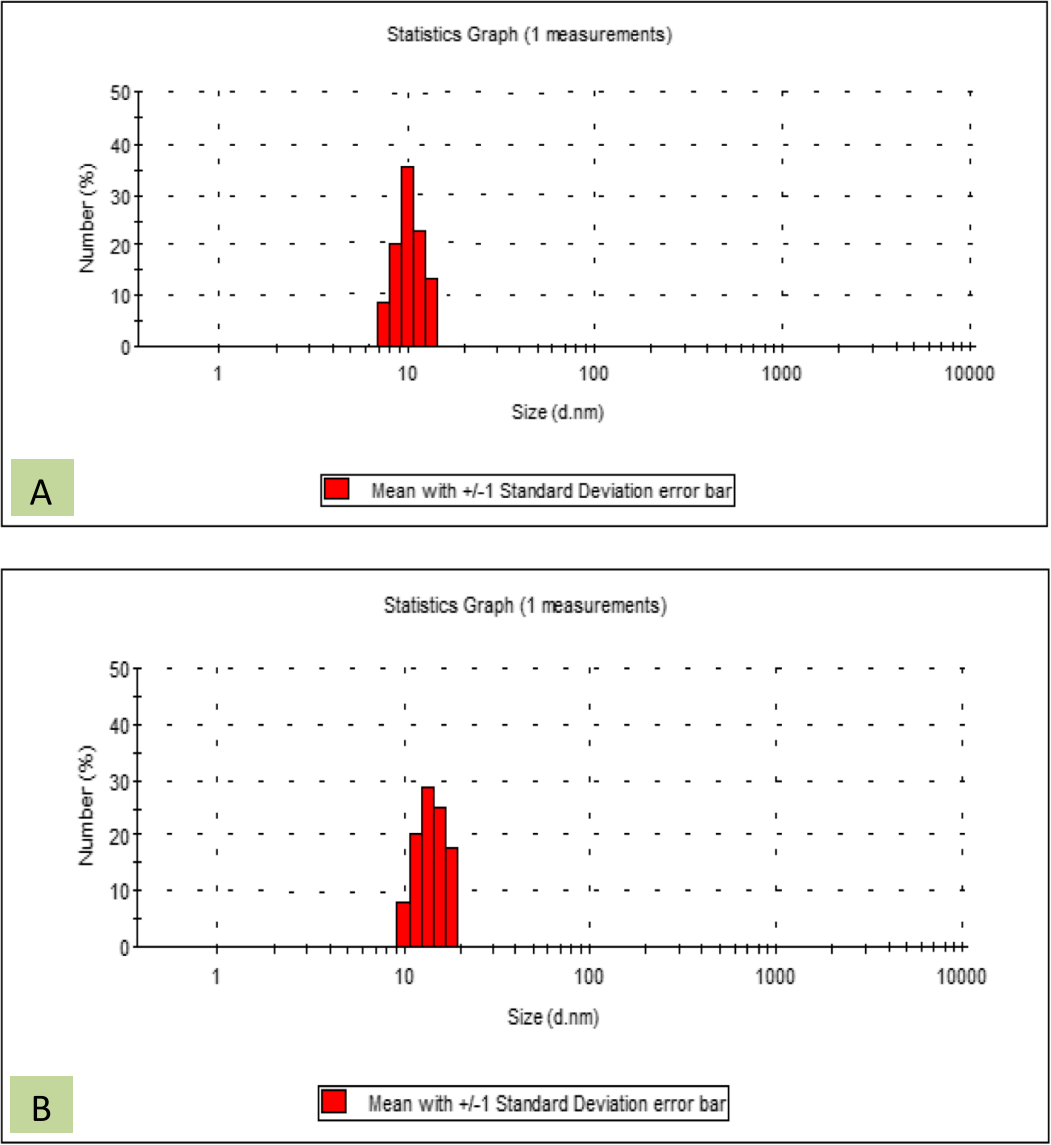
The size and size distribution of (**a**) Iron oxide nanoparticles (Fe3O4 NPs) and (**b**) Genistein conjugated Fe3O4-CMC nanoparticles according to DLS results

The size of nanoparticles (NPs) was assessed by dynamic light scattering (DLS). Iron oxide MNPs have various size of between 7 to 14 nm. Figure 3 indicated that the Fe_3_O_4_ nanoparticles exhibit a narrow size distribution and the mean diameter of these particles is calculated around 10 nm. After conjugation of Fe_3_O_4_-CMC with genistein, the size of nanoparticles increased to 8 to 16 nm.

### Crystallography and phase study

**Fig. 4:**
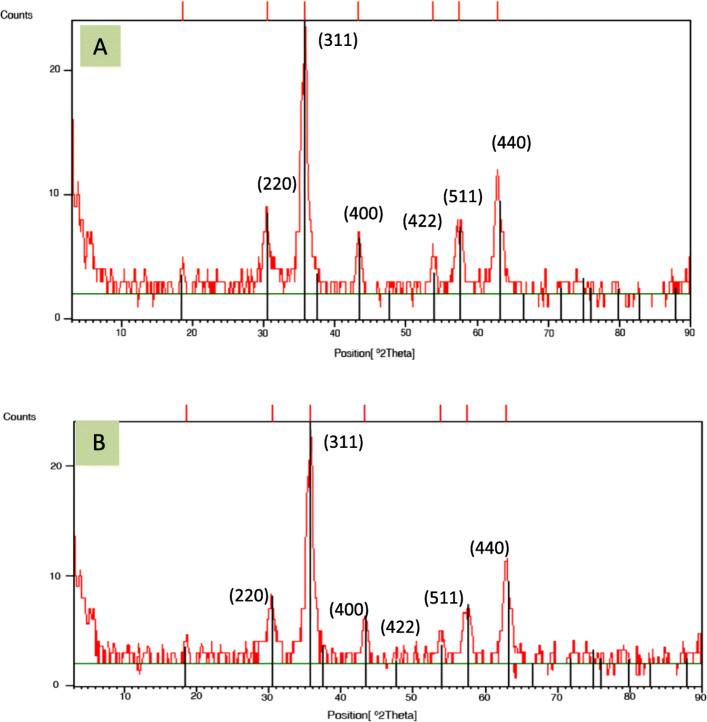
X-ray diffraction patterns for (**a**) Iron oxide nanoparticles (Fe3O4 NPs) and (**b**) Genistein conjugated Fe3O4-CMC nanoparticles

The pattern of X-ray diffraction of naked iron oxide NPs, Fe_3_O_4_-CMC NPs and Fe_3_O_4_-CMC-genistein NPs are shown in Fig. 4. Naked iron NPs show six intense peaks between 30° and 70° at 30.3854°, 35.6762°, 43.3200°, 53.8317°, 57.4892°and 62.9182°, which are attributed to (220), (311), (400), (422), (511), and (440). It confirmed that the magnetite NPs were pure iron oxide with a cubic inverse spinal structure by this method. Pure iron oxide NPs crystallite size calculated by Debye-Scherrer eq. (D = Kλ/βcosθ). In this equation, K is the Debye-Scherrer constant (0.9), λ is the X-ray wavelength (1.54178 nm), β is the peak width of half-maximum, and θ is the diffraction angle [24]. The crystallite size was about 8.8 nm.

### The surface reactions

**Fig. 5:**
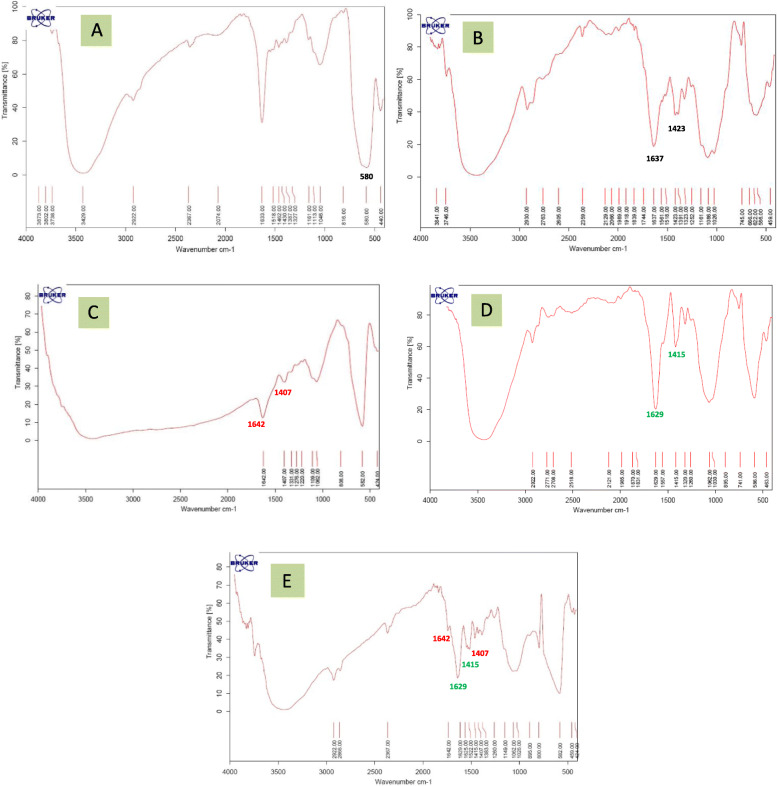
Fourier transform infrared spectra for (**a**) Iron oxide nanoparticles (Fe3O4 NPs), **b** Carboxymethyl chitosan, **c** Genistein, **d** Chitosan coated iron oxide nanoparticles, **e** Genistein conjugated Fe3O4-CMC nanoparticles

Fourier transform infrared spectroscopy (FTIR) and spectra of Fe_3_O_4_ nanoparticles of CMC and Fe_3_O_4_-CMC nanoparticles before conjugation with genistein and also spectra of naked genistein and Fe_3_O_4_-CMC-genistein are shown in Fig. 5. In this figure, intense and broad band in the 3200–3600 cm^− 1^ of the spectrum of Fe_3_O_4_nanoparticles confirms that NPs surface is covered with hydroxyl groups. Also, the vibration band, which is seen in about 560–580 cm^− 1^ is characteristic band for naked iron oxide NPs and is attributed to Fe-O group [25] (Fig. 5a).

The surface chemistry and the spectra of CMC shows the characteristic band at around 3425 cm^− 1^ corresponding to the O − H stretching and N-H stretching vibrations. The peaks at 1637 cm^− 1^ and 1423 cm^− 1^ are related to the N–H bend vibration of amines functional groups of CMC and symmetric stretching vibration of COO, respectively (Fig. 5b). In the spectra of Fe_3_O_4_-CMC nanoparticles, the main absorption peaks are at 1629 cm^− 1^ and 1415 cm^− 1^ wave numbers. Descending of 1637 cm^− 1^ and 1423 cm^− 1^ to lower wave numbers of 1629 cm^− 1^ and 1415 cm^− 1^ can be related to the successful bonding between the hydroxyl groups of the Fe_3_O_4_ nanoparticles and the carboxyl groups of CMC [26] (Fig. 5d). Figure 5e shows the spectra of Fe_3_O_4_-CMC-genistein nanoparticles. The spectrum of the resulted Fe_3_O_4_-CMC-genistein nano-conjugate shows the characteristic bands of the original Fe_3_O_4_-CMC at 1629, 1415 cm^− 1^ and also characteristic peaks of genistein were at 1407 and 1642 cm^− 1^ (Fig. 5c) confirming that genistein is successfully conjugated onto the Fe_3_O_4_-CMC nanoparticles.

### Magnetic properties

**Fig. 6:**
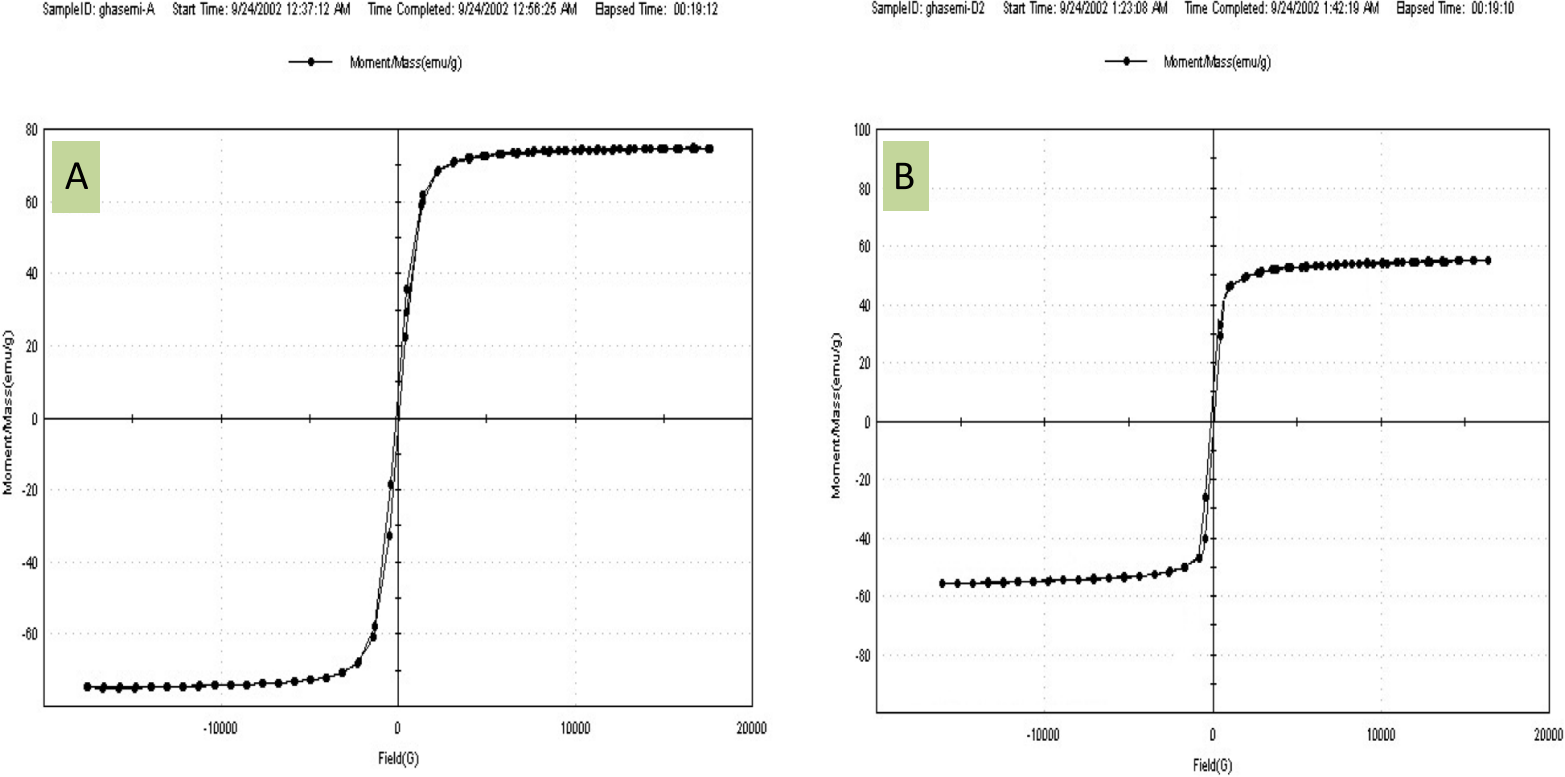
Hysteresis loop for (**a**) Iron oxide nanoparticles (Fe3O4 NPs), (**b**) Genistein conjugated Fe3O4-CMC nanoparticles

The synthesized nanoparticles of genistein were analyzed by using a vibrating sample magnetometer at room temperature. The hysteresis loops for samples are shown in Fig. 6. The saturation magnetization for the Fe_3_O_4_ and Fe_3_O_4_-CMC-genistein nanoparticles was 74.85 emu/g and 59.60 emu/g respectively.

**Fig. 7:**
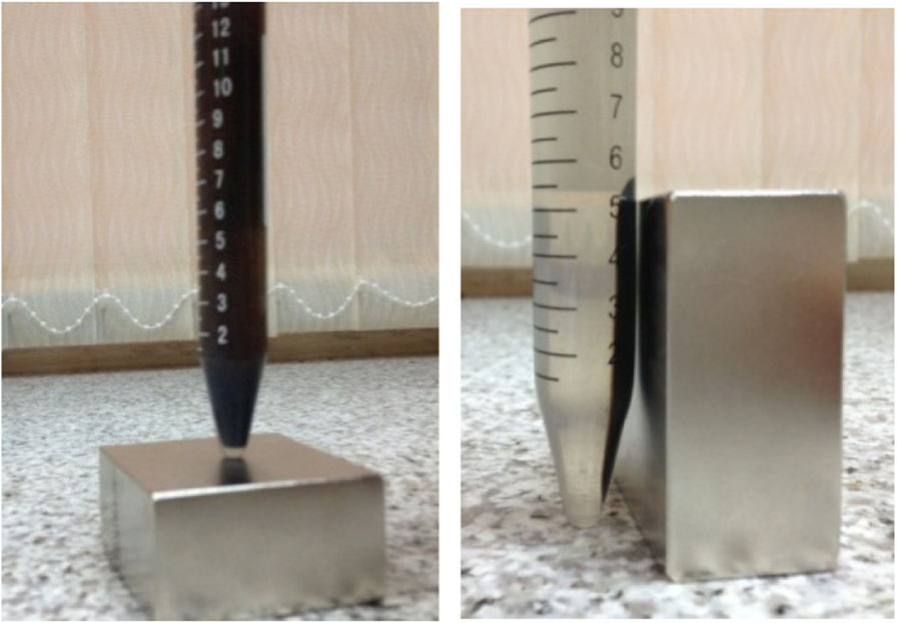
Separation of magnetic nanoparticles under an external magnetic field

The large magnetic particles aggregate after exposure to a magnetic field. Synthesizing smaller size nanoparticles with high degree of magnetization can offer suitable properties in biomedical applications, which is our aim in this research work. As it can be seen in the Fig. 7, particles can be easily separated from the solution in the presence of magnetic field. Our samples show super-paramagnetic behaviors. It demonstrated that magnetite nanoparticles were incorporated in the composite particles with no remanence effect from the hysteresis loops when magnetic field applies.

### Cell growth studies

**Fig. 8:**
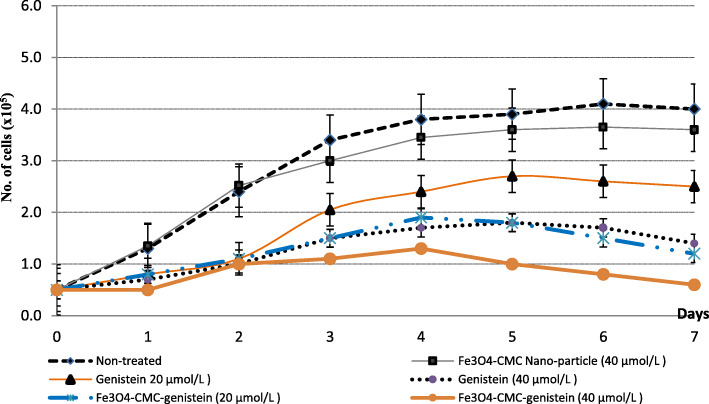
Cell growth assay of JURKET in the effect of 20 and 40 μmol/L genistein as well as 20 and 40 μmol/L Fe3O4-CMC-genistein as compared to non-treated cells as well as treated cells with 40 μmol/L only Fe3O4-CMC nano-particles

The cell lines of acute lymphoblastic lymphoma (ALL) were treated to naked genistein as well as synthesized nano-conjugated Fe_3_O_4_-CMC-genisteinin. After a single treatment at 72th hours with 25 μmol/L and 50 μmol/L genistein as well as nano-conjugated genistein, viable cells were counted for each 24 h intervals. Consequently, 7-days growth curves were obtained in order to evaluation of long term survival rate (Fig. 8).

We observed that cell growth of these ALL cell lines were reduced to as low as 31.0% compared with genistein-treated cells in the effect of 20 μmol/L nano-conjugated genistein. Abatement of cell growth around 34% was took place by 20 μmol/L genistein, whereas this decreasing for 54% for 20 μmol/L nano-conjugated Fe_3_O_4_-CMC-genistein, as comparison with non-treated cells. The same trait was observed for higher concentration (40 μmol/L). Decreasing of cell growth by applying 40 μmol/L nano-conjugated Fe_3_O_4_-CMC-genistein was 33% lower than 50 μmol/L naked genistein. In spite of higher effectiveness of 50 μmol/L nano-conjugated genistein in reduction of cell growth in comparison with 20 μmol/L nano-conjugated Fe_3_O_4_-CMC-genistein, but there was no significant difference observed (*p* = 0.55). Interestingly, the reduction of cancer cell growth induced by 20 μmol/ Fe_3_O_4_-CMC-genistein nano-conjugate was similar to 40 μmol/L genistein (Fig. 8). It seems that nano-conjugation of genistein with Fe_3_O_4_-CMC could avoid the usage of higher concentration of genistein. It seems that nano-conjugation of genistein can improve its chemotherapy against of ALL cancer even in lower doses near to physiological serum level. It expects to minimize side effects such as toxicity on normal cells.

**Fig. 9:**
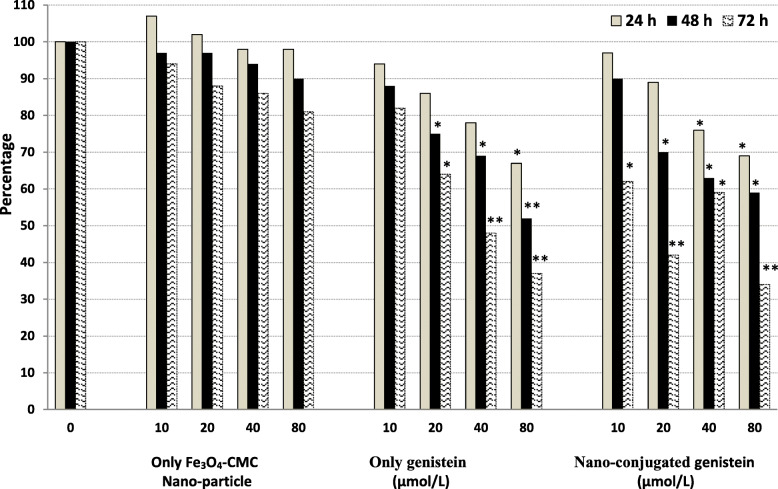
Proliferating assay of JURKET cells after treating by genistein & nano-conjugated genistein in different concentration 10, 20, 40, 80 μmol/L during 24, 48 and 72 h. Results stated in percentage change in comparison with the zero concentration of genistein. * indicates *p* < 0.05 and ** refers to *p* < 0.005

We also treated hematopoietic cancer cells with concentrations of 20, 40, 60, 80 and 100 μmol/L of genistein and Fe_3_O_4_-CMC-genistein nano-conjugation for 24, 48 and 72 h. The effect of genistein and its synthesized nanoparticles on cell proliferation was also evaluated by MTT assay. Obtained findings through MTT assay indicated that both genistein and nano-conjugated Fe_3_O_4_-CMC-genistein could significantly reduce proliferation of cells (*p* < 0.001), but nano-conjugated genistein totally showed higher inhibition rate in comparison with genistein, especially in 72 h. Treatment of hematopoietic cancer cells with nano-conjugated at higher doses (80 and 100 μM) for 24 h had lower inhibition effect than naked genistein. The inhibition effect of genistein was even little better than the effect of nano-conjugate genistein after 48 h of treatment. Reduction of cell proliferation for 44% was observed by 72 h treatment of nano-conjugated Fe_3_O_4_-CMC-genistein in comparison with naked genistein (Fig. 9). The prevention of cell proliferation in effect of 10 to 80 μmol/L of nanoparticles calculated from 34.0 to 71.6% in MTT assay after 72 h treatment.

### Apoptosis analysis

The percentage of apoptosis has been analyzed by FACS analysis in the effect of 48 h treatment with 20 and 40 μmol/L genistein, 20 and 40 μmol/L nano-conjugated Fe_3_O_4_-CMC-genistein and 40 μmol/L Fe_3_O_4_-CMC nano-particles as compared to non-treated JURKAT cell lines.

**Fig. 10:**
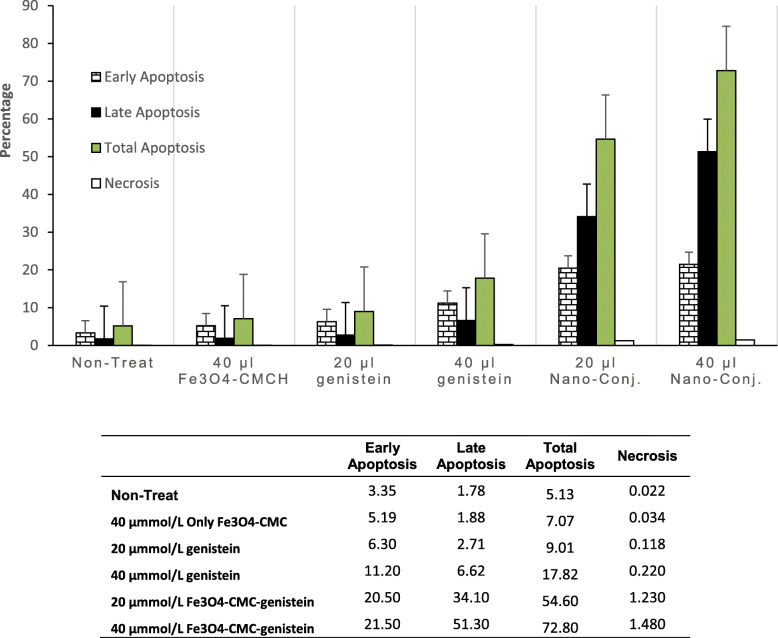
The impact of 48 h treatment with different dose of Genistein on Apoptosis of JURKET cell line obtained by Flow Cytometry (The Flow Cytometry charts supply in supplementary file)

The FACS analysis showed an increase in the percentage of early apoptotic cells from 5.1 to 11.2% for dose of 20 and 40 μmol/L of treated cells with only genistein, but 20.5 and 21.5% cells were on early apoptotic stages in effect of 48 h treatment with 20 and 40 μmol/L nano-conjugated Fe_3_O_4_-CMC-genistein (Fig. 10). Significant difference of early apoptotic cells was observed between 10 and 20 μmol/L of nano-conjugated Fe_3_O_4_-CMC-genistein and 20 and 40 μmol/L of only genistein (*p* = 0.012). No significant increase was observed in apoptosis of cells treated with 40 μmol/L nano-particles contained only Fe_3_O_4_-CMC in comparison with non-treated cells (*p* = 0.114). Also, no significant difference in percentage of early apoptotic cells was observed in 24 h and 48 h treatment with nano-conjugated Fe_3_O_4_-CMC-genistein (*p* = 0.064) as well as 20 and 40 μmol/L treatment (*p* = 0.079).

## Discussion

The synthesizing procedure designed for conjugation of genistein to iron oxide nanoparticles coated with biocompatible cross-linked chitosan, which is schematically illustrated in Fig. 1. Carboxic groups existed in carboxymethylated chitosan (CMC) supply active sites for binding genistein and increase water-solubility of nanoparticles [12]. EDC provide condensation circumstance for reaction carboxyl groups with –OH or –NH_2_ groups. In fact, EDC activated carboxyl group of CMC to form an intermediate reactive ester, which covalently react with -OH groups on the Fe_3_O_4_ nanoparticles to decorate superparamagnetic nanoparticles with CMC, and also “induce cross-linking between chitosan” [12, 27]. Then, genistein was attached to Fe_3_O_4_-CMC nanoparticles in circumstance provided by EDC condensation reaction to achieve final Fe_3_O_4_-CMC-genistein nano-conjugation for drug delivery system.

Obtained data from transmission electron microscopy (TEM) showed that spherical shape of Fe_3_O_4_ and Fe_3_O_4_-CMC-genistein nanoparticles with distance from each other indicated their good water solubility [12]. Also, iron oxide MNPs tend to aggregate due to their magnetization effect, high surface energy and high surface area [28]. A mean diameter around 12ƞm also confirmed proper synthesis of these particles. By compare the X-ray diffraction of three nanoparticles of naked iron oxide, Fe_3_O_4_-CMC and Fe_3_O_4_-CMC-genistein showed that the surface functionalization did not cause any phase change to the Fe3O4 nanoparticles and the crystal structures of samples are the same. The obtained data from fourier transform infrared spectroscopy (FTIR) confirmed that not only NPs surface is covered with hydroxyl groups but also genistein successfully conjugated onto the Fe_3_O_4_-CMC nanoparticles.

In the other hand, the high amount of saturation magnetization of naked Fe_3_O_4_ MNPs, although is smaller than the bulk Iron oxide (around 92 emu/g), indicate the good crystal structure [28]. The saturation magnetization of Fe_3_O_4_-CMC-genistein NPs was smaller than the value for the naked Fe_3_O_4_. The reduction of saturation magnetization showed that the Fe_3_O_4_ MNPs are surrounded by organic materials [29]. Decrease of the mass saturation magnetization in the component maybe due to the surface functionalization, which caused the linkage between CMC and genistein onto the surface of Fe_3_O_4_ nanoparticles [30]. Our synthesized nano-particles incorporated in the composite particles also demonstrated no remanence effect from the hysteresis loops when magnetic field applies.

The anti-cancer activity of synthesized Fe_3_O_4_-CMC-genistein is dose- and time-dependent. These observations were also reported by other researchers such as Wang et al., (2006) [31] and Choi et al., (2007) [32]. It could be directed to a slow release of genistein from the nano-conjugated Fe_3_O_4_-CMC-genistein. As the genistein molecules were bounded covalently to the Fe_3_O_4_ nanoparticles, the prohibition effect might be weak at once. The ester bond between genistein and the carboxymethyl chitosan could be hydrolyzed and genistein started to release increasingly, resulted to augment prohibit effect by passing the time. This result was similar with observations of Si et al., (2010) on gastric cancer cell line [12].

Annexin based FACS analysis shows rate of apoptosis and MTT assay indicate cell proliferation. Induction of apoptosis is one of major mechanism for growth inhibition of hematopoietic cancer cell lines by nano-conjugated Fe_3_O_4_-CMC-genistein in lower dose. Induction of early apoptosis by free drug was distinctly lower than nano-particles. In collation of obtained data by FACS analysis and MTT assay, it deduced that genistein can induce its anticancer effect by inhibition of proliferation and induce of apoptosis. It was also found that genistein in form of this synthesized nano-particle induce its effect continuously, long lasting and more effective for two times in lower dose for half.

## Conclusions

In this study, we examined the effect of iron oxide MNPs in combination with genistein for increasing its anticancer effects. We have prepared a drug delivery system for genistein using iron oxide MNPs and CMC. A suitable polymer for coating iron oxide MNPs is CMC due to its potential to create links between Fe_3_O_4_ NPs and genistein. Another prominent characteristic for CMC is its water solubility. The covalent binding between Fe_3_O_4_-CMC NPs and genistein leads to design such a biocompatible drug delivery vehicle. By using EDC as a strong cross linker, these bindings can be formed. The results were confirmed by using TEM, DLS, FTIR, XRD and VSM analysis. The analysis showed that the size of nanoparticles increases after coating with CMC and linking with genistein whereas in each step by increasing the size of particles, the magnetic properties decrease, which indicates proper binding with CMC and genistein.

MTT assay and annexin-based FACS flow cytometry was used for determination of the effectiveness of binding genistein in this drug delivery system. This assay shows that the genistein was slowly released from the Fe_3_O_4_-CMC-genistein drug delivery system to hematopoietic cancer cells and its effect will probably long-last and pushed the cancer cells toward apoptosis. The synthesized Fe_3_O_4_-CMC-genistein nano-conjugated cause to empower its anti-cancer activity in lower dose. This can probably improve its chemotherapeutic features because of reduction of its toxicity and increase of effectiveness in lower dose. Fe_3_O_4_-CMC-genistein drug delivery system could be considered as promising and futuristic carrier for natural compounds with anticancer effect such as genistein. Since very few drug carriers included both chitosan and magnetic nanoparticles have been reported, particularly on genistein, hence further study is necessity on other cancer cell lines as well as animal models.

## Supplementary information

**Additional file 1. **The FACS result of 48 h treatment with different dose of Genistein on Apoptosis of JURKET cell line obtained by Flow Cytometry; **(i)** concentration zero (as control); **(ii)** 40 μmmol/L only Fe3O4-CMCH without conjugated genistein; **(iii)** 20 and **(iv)** 40 μmmol/L only genistein; **(v)** 20 and **(vi)** 40 μmmol/L Fe3O4-CMCH-genistein nano-conjugate.

## Data Availability

All data generated and analyzed during the current study are available from the corresponding author on reasonable request.

## Abbreviations

ALL: Acute Leukemia Lymphoma
CMC: Carboxymethylated chitosan
DLS: Dynamic light scattering
DMSO: Dimethyl sulphoxide
FBS: Fetal bovine serum
FTIR: Fourier-transform infrared spectroscopy
MNPs: Magnetic nanoparticles
MTT: Thiazolyl Blue Tetrazolium Bromide
NHS: N-Hydroxysuccinimide
NPs: Nanoparticles
OCMCS: O-carboxymethyl chitosan
Penstrep: Penicillin /Streptomycin
PI: Propidium Iodide Solution
SPIONs: Superparamagnetic iron oxide nanoparticles
TEM: Transmission electron microscopy
VSM: Vibrating-sample magnetometer
XRD: X-ray powder diffraction
