# Supplementary material for: Synthesizing efficacious genistein in conjugation with superparamagnetic Fe3O4 decorated with bio-compatible carboxymethylated chitosan against acute leukemia lymphoma
Source: Biomater Res. 2020 Mar 20;24:9. doi: 10.1186/s40824-020-00187-2 (PMC7082912; doi:10.1186/s40824-020-00187-2)
Supplement: Supplementary file 1 — Additional file 1. The FACS result of 48 h treatment with different dose of Genistein on Apoptosis of JURKET cell line obtained by Flow Cytometry; (i) concentration zero (as control); (ii) 40 μmmol/L only Fe3O4-CMCH without conjugated genistein; (iii) 20 and (iv) 40 μmmol/L only genistein; (v) 20 and (vi) 40 μmmol/L Fe3O4-CMCH-genistein nano-conjugate. [file 40824_2020_187_MOESM1_ESM.docx]

**Fig.10 supplementary:**

The FACS result of 48 hr treatment with different dose of Genistein on Apoptosis of JURKET cell line obtained by Flow Cytometry; **(i)** concentration zero (as control); **(ii)** 40 µmmol/L only Fe3O4-CMCH without conjugated genistein; **(iii)** 20 and **(iv)** 40 µmmol/L only genistein; **(v)** 20 and **(vi)** 40 µmmol/L Fe3O4-CMCH-genistein nano-conjugate.


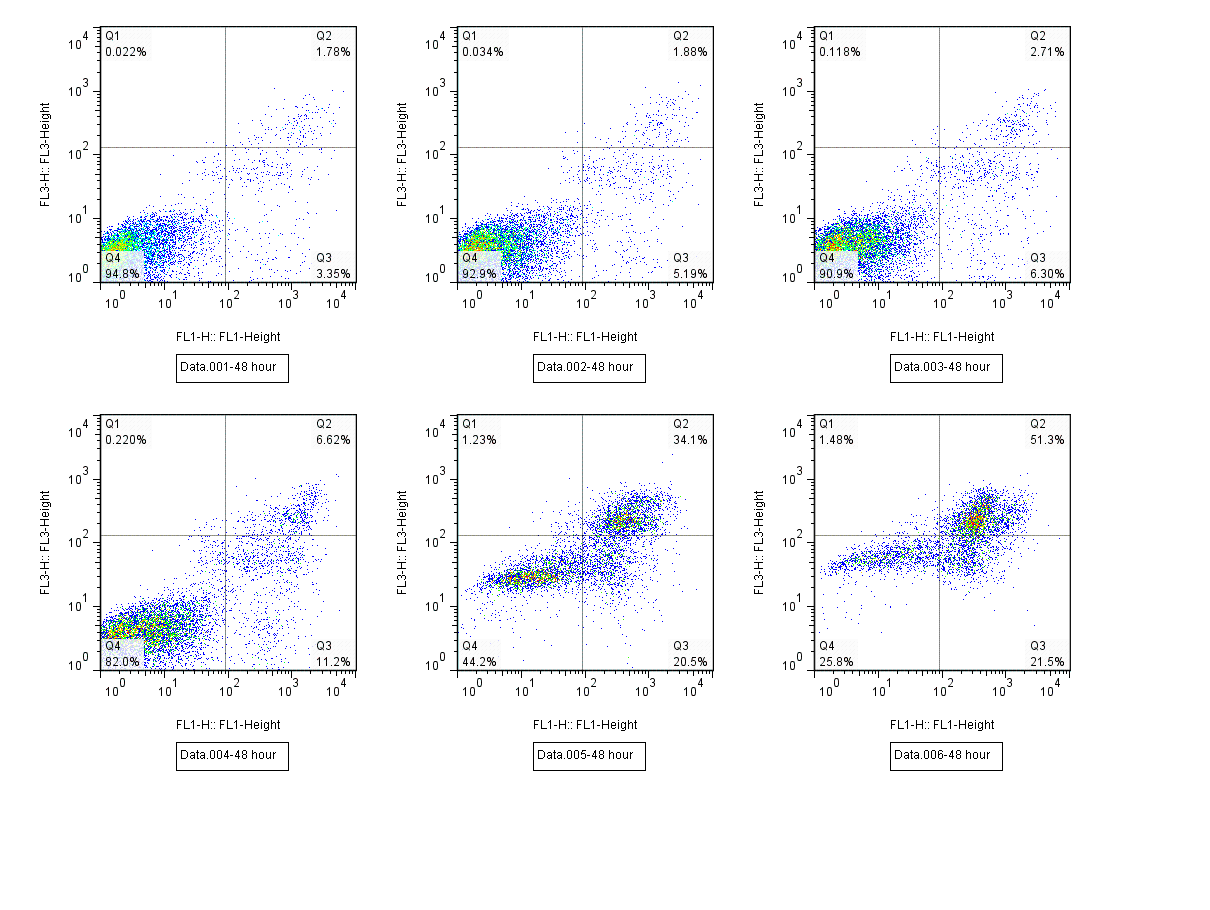


**i**

**ii**

**iii**

**iv**

**v**

**vi**
